# Comparison of the molecular profiles of human embryonic and induced pluripotent stem cells of isogenic origin

**DOI:** 10.1016/j.scr.2013.11.010

**Published:** 2013-12-04

**Authors:** Barbara S. Mallon, Rebecca S. Hamilton, Olga A. Kozhich, Kory R. Johnson, Yang C. Fann, Mahendra S. Rao, Pamela G. Robey

**Affiliations:** a NIH Stem Cell Unit, National Institute of Neurological Disorders and Stroke, National Institutes of Health, Bethesda, MD 20892, USA; b Laboratory of Molecular Biology, National Institute of Neurological Disorders and Stroke, National Institutes of Health, Bethesda, MD 20892, USA; c Bioinformatics Section, Intramural IT and Bioinformatics Program, National Institute of Neurological Disorders and Stroke, National Institutes of Health, Bethesda, MD 20892, USA; d NIH Center for Regenerative Medicine, National Institutes of Health, Bethesda, MD 20892, USA; e Craniofacial and Skeletal Disease Branch, National Institute of Dental and Craniofacial Research, National Institutes of Health, Bethesda, MD 20892, USA

## Abstract

Many studies have compared the genetic and epigenetic profiles of human induced pluripotent stem cells (hiPSCs) to human embryonic stem cells (hESCs) and yet the picture remains unclear. To address this, we derived a population of neural precursor cells (NPCs) from the H1 (WA01) hESC line and generated isogenic iPSC lines by reprogramming. The gene expression and methylation profile of three lines were compared to the parental line and intermediate NPC population. We found no gene probe with expression that differed significantly between hESC and iPSC samples under undifferentiated or differentiated conditions. Analysis of the global methylation pattern also showed no significant difference between the two PSC populations. Both undifferentiated populations were distinctly different from the intermediate NPC population in both gene expression and methylation profiles. One point to note is that H1 is a male line and so extrapolation to female lines should be cautioned. However, these data confirm our previous findings that there are no significant differences between hESCs and hiPSCs at the gene expression or methylation level.

## Introduction

Human induced pluripotent stem cells (hiPSCs) share key features and potential of human embryonic stem cells (hESCs) and allow the generation of patient-specific material (Ebert et al., 2009; Soldner et al., 2009). However, the extent to which they faithfully recapitulate the characteristics of embryonic stem cells remains a subject of debate (Feng et al.,2010; Hu etal., 2010; Smith et al., 2009). There have been multiple studies in recent years comparing gene expression and methylation profiles of ESCs and iPSCs (Bock et al., 2011; Chin et al., 2009; Lister et al., 2011; Mallon et al., 2013) and a number of studies have shown evidence that generation of iPSCs can induce abnormalities at both genetic and epigenetic levels (Gore et al., 2011; Hussein et al., 2011; Laurent et al., 2011; Lister et al., 2011; Mayshar et al., 2010). In addition, there has been much made of ‘epigenetic memory’ in which induced pluripotent cells are said to retain some epigenetic marks of the donor cell type from which they were derived (Kim et al., 2010; Marchetto et al., 2009).

Previously, we reported that there were no significant gene expression differences between 21 hESCs and 8 hiPSCs (Mallon et al., 2013) in accordance with other findings (Guenther et al., 2010). In that study, we found that, although some genes were variably expressed, there were no genes that were significantly increased in one population over the other. Although some studies have described differences in the methylation profile between hESCs and hiPSCs (Bock et al., 2011; Deng et al., 2009; Doi et al., 2009; Kim et al., 2010; Lister et al., 2009), this may simply reflect normal human variation (Lo et al., 2003; Yan et al., 2002) or may actually be a result of the reprogramming process. To address this, Teichroeb et al., compared the genetic profile of H9 (WA09) hESCs to a clonally purified mortal splanchnopleuric mesodermal somatic cell line differentiated from them, EN13, and hiPSCs derived from these differentiated cells (Teichroeb et al., 2011). In this female line, they found the gene expression profiles to be generally very similar with the only striking difference in gene expression being that of neuronatin (*NNAT*), a maternally silenced imprinted gene. This, together with their observation of hotspots of de-repression of X chromosome inactivation (XCI), led them to conclude that reprogramming affects both imprinting and XCI. However, a recent review of this phenomenon indicated that much of this genetic and epigenetic drift may be caused by local culture conditions (Wutz, 2012).

To complement and extend the previous study, we have used a well known male hESC line, H1 (WA01), to investigate the gene expression and methylation profiles of isogenic hESCs and hiPSCs. We derived an intermediate neural stem cell precursor cell (NPC) line from these hESCs (Kozhich et al., 2012) and expanded it several times before reprogramming to induced pluripotent stem cells using lentiviral transduction of the 4 Yamanaka factors (Oct4, KLF4, SOX2 and c-myc). Several independent colonies were expanded and analyzed for gene expression and methylation using array-based analyses as described previously (Mallon et al., 2013). Although the iPSC lines derived vary slightly at the genomic level due to lentiviral integration, in accordance with the previous study (Teichroeb et al., 2011), these lines are referred to in this manuscript as isogenic reflecting the overall similarities between them and the parental H1 line. No significant difference in gene expression was found between the pluripotent cell populations under undifferentiated or differentiated conditions. Similarly, little variation was observed in the methylation profile of the undifferentiated cells although some deviation was observed between the pluripotent populations and the NPCs as would be expected following differentiation. Examination of the gene expression of putative imprinted genes showed variability in expression for many of them, often in response to differentiation. However, no genes were expressed at significantly different levels in the isogenic hiPSC population relative to the H1 parental line. We more closely examined *NNAT* expression in the StemCellDB database and found that gene expression was variable in both hESC and hiPSC populations and appeared to be regulated by methylation. Interestingly, the hiPSCs appeared to be more sensitive to down-regulation by increased methylation. However, this phenomenon was not apparent in the current H1 isogenic study. All microarray and methylation array data may be accessed through the NCBI GEO public database (Superseries number GSE51748).

## Experimental procedures

### Feeder-based pluripotent stem cell culture

All culture reagents were acquired from Life Technologies unless stated otherwise. Standard culture conditions of 37 °C, 5% CO_2_ and 95% humidity were maintained for all cells.

Human pluripotent stem cells (hPSCs) were cultured on a feeder-layer of irradiated CF1 mouse embryonic fibro-blasts (MEFs) in DMEM: F12 (Cat# 11330-032) containing 20% Knockout Serum Replacement (KSR), 1 mM glutamine, 0.1 mM β-mercaptoethanol (β-ME; Sigma), 1× non-essential amino acids (NEAA) and 4 ng/ml bFGF (R&D Systems). Fibroblasts were cultured in DMEM (Cat# 11965-092) containing 10% fetal bovine serum (FBS) (Gemini Bio-products), 2 mM glutamine and 1× NEAA. Fibroblasts were irradiated with ~6500 rads using a Faxitron RX650 X-irradiator. They were subsequently plated on Falcon 6-well tissue culture dishes, coated with 0.1% gelatin, at a density of 0.1875 × 10^6^/well. hPSCs were plated in small clumps of approximately 100 cells the following day, medium was exchanged every day and colonies were passaged by collagenase treatment every 3–4 days. Briefly, cultures were treated with 1.5 mg/ml collagenase IV for 20–40 min and either tapped sharply or scraped to dislodge colonies. Colonies were allowed to sediment for 5 min, the supernatant was removed and fresh media added. This process was repeated for a total of 3 sediments. At this point cells were triturated to generate colonies of approximately 10–100 cells for passaging.

### Derivation of neural precursor cells (NPCs) from H1 (WA01) human embryonic stem cells

A proliferating population of neuronal precursor cells (NPCs) were derived from the H1 (WA01) human embryonic stem cell line as previously described (Kozhich et al., 2012). Briefly, embryoid bodies (EBs) were cultured in DMEM containing B27 minus Vitamin A and 2 mM l-glutamine, (Neural Precursor Medium; NPM) supplemented with 500 ng/ml noggin (R&D Systems) and 20 ng/ml bFGF (R&D Systems) for 2 weeks. At this time the EBs were plated out in a minimal medium consisting of DMEM containing an Insulin–Transferrin–Selenium supplement (Life Technologies), 2 mM l-glutamine and 5 ng/ml human fibronectin (Roche) for 1 week. Migrating neuroepithelial cells were isolated by Accutase (Innovative Technologies Inc.) treatment and differential sedimentation to remove the larger aggregates. These NPCs were propagated on polyOrnithine/laminin (both Sigma) coated plates in NPM supplemented with 20 ng/ml each of bFGF and EGF (R&D Systems).

### Reprogramming

A slight modification of the manufacturer’s instructions for the StemCCA kit (Millipore) was followed. Briefly, approximately 40,000 NPCs were plated overnight in a 48-well plate coated with poly-l-ornithine and laminin. An amount of virus appropriate to give a MOI of 200 was added to one well of H1.NPCs along with 5 μg/ml polybrene in NPM containing bFGF and EGF. The next day, cells were washed once with Dulbecco’s phosphate buffered saline (d-PBS) and the transduction was repeated. Cells were washed three times with D-PBS and NPM containing bFGF and EGF as well as chemicals 5 μM PS48 (Reagents Direct), 0.5 μM A-83-01 (StemGent) and 0.25 mM sodium butyrate (StemGent) to enhance reprogramming (Zhu et al., 2010), was added. After 6 days of culture, the cells were dissociated with Accutase and replated on 1 well of MEFs in NPM containing bFGF and EGF. The day after plating the medium was switched to hESC culture medium containing the reprogramming cocktail and cells were fed daily with this medium until colonies started to appear. At that time reprogramming chemicals were omitted and cells were fed with standard hESC medium. After approximately 3 weeks, visible iPSC colonies were individually isolated and expanded. The remaining colonies on the well were passaged with collagenase and replated on Matrigel in mTeSR1 as described below.

### Feeder-free pluripotent stem cell culture

To coat 6-well plates with Matrigel, 1.5 ml of 1.25% Matrigel in DMEM:F12 was added to each well and incubated at 4 °C overnight. The plate was allowed to warm to room temperature prior to plating small clumps as described above in mTeSR1 medium (Stem Cell Technologies). Colonies were maintained in mTeSR1 medium and passaged as described for feeder-based culture except for the use of 1 mg/ml dispase instead of collagenase IV.

### EB formation

Embryoid bodies were formed by culturing detached colonies in fibroblast medium (EB_mesend) or in hESC medium without bFGF (EB_ecto) in 60 mm Corning Low Attachment dishes for a total of 8 days. Media were changed by sedimentation every 2 days.

### FACS analysis

After collection of the colonies by collagenase or dispase treatment as described above, cells were washed once with Dulbecco’s phosphate buffered saline (D-PBS) and treated with Accutase for 10 min at 37 °C. Cells were washed with 5 ml DMEM:F12, centrifuged at 200 g for 5 min and resuspended in FACS buffer (10% FBS in DMEM:F12) and maintained on ice. Antibodies, including IgM isotype control (Sigma), were diluted to twice the final concentration listed below and 50 μl of each dispensed into a well of a round-bottom 96-well plate (Fisher Scientific). To this was added 50 μl of cell suspension containing approximately 10–25 × 10^4^ cells. Antibodies used were SSEA4 (1:100; Santa Cruz sc-21704), Tra-1-60 (1:100; Santa Cruz sc-21705) and NCAM (1:250; Millipore Corp MAB5324). Cells were incubated for 1 h at 4 °C, washed with FACS buffer and incubated with 1:100 AlexaFluor 488-conjugated anti-IgM (Life Technologies) for 30 min at 4 °C. Cells were again washed with FACS buffer and resuspended in approximately 125 μl FACS buffer prior to FACS analysis using a Becton Dickenson FACSCalibur with a 96-well plate HTS attachment. Data was collected and analyzed using Plate Manager and CellQuest Pro software (BD).

### Negative magnetic sorting

Cells were subjected to negative magnetic sorting using Tra-1-60 antibody and IgM conjugated magnetic beads (Miltenyi Biotech) according to the manufacturer’s instructions.

### Immunostaining

Cells were fixed by rinsing briefly with D-PBS followed by 20 min incubation with 4% paraformaldehyde in PBS (Electron Microscopy Sciences) at room temperature. Cells were blocked and permeabilized by incubation for 40 min in 10% normal goat serum (NGS; Sigma) containing 0.3% Triton X-100 (Sigma) in D-PBS. Appropriate dilutions of primary antibodies were made in 5% NGS in D-PBS and applied to cells after 3 washes in D-PBS (1:500 Oct3/4, Santa Cruz sc-5279; 1:5000 albumin, Cedarlane CL2513A; 1:100 AFP, Santa Cruz sc-8399; 1:100 HNF4A, Cell Signaling Technologies 3113S; 1:1000 TuJ1, Covance MMS-435P; 1:100 nestin, Millipore MAB5326; 1:200 MAP-2, Sigma M1406; 1:40 Myosin Heavy Chain β, MAB1548; 1:200 Troponin T, Lab Vision Corporation MS-295-PO). After 3 h incubation at RT, cells were washed and incubated with 1:1000 dilutions of appropriate AlexaFluor-conjugated secondary antibodies in 5% NGS in D-PBS (Life Technologies). Cells were incubated with secondary antibodies for 1 h, washed 3 times in D-PBS and incubated with 20 ng/ml bis-benzimide (Hoechst dye, Sigma Cat# B-1155) for 20 min. After washing, cells were mounted using AquaPolymount (Fisher) and imaged using a Retiga camera, Zeiss Axiovert 200 inverted fluorescent microscope and QCapture/Photoshop software.

### Directed differentiation to endoderm and mesoderm

Cells were directed toward an endodermal (hepatocyte) fate using a slight modification of the method of Si-Tayeb et al. (Si-Tayeb et al., 2010). Briefly, pluripotent stem cells were cultured on Matrigel for 3 days in mTeSR1 medium before switching the medium to RPMI (Mediatech) supplemented with B27 (Life Technologies) and 100 ng/ml Activin A (R&D Systems). After 5 days the medium was switched to RPMI supplemented with B27, 20 ng/ml BMP-4 and 10 ng/ml bFGF (R&D Systems). Five days later the medium was again switched to RPMI supplemented with B27 and 20 ng/ml HGF (R&D Systems). In the original paper (Si-Tayeb et al., 2010) these 2 steps were performed at hypoxic (4%) oxygen concentrations but were performed here at normoxic concentrations (20%). After a further five days the medium was again switched to Hepatocyte Culture Medium (Lonza) supplemented with 20 ng/ml oncostatin M (Millipore Corp). Cells were incubated for 5 days prior to fixation. During each stage the medium was exchanged once.

Cells were directed to a mesodermal (cardiomyocyte) fate using a slight modification of a protocol by He et al. (He et al., 2003). Colonies of pluripotent cells, cultured on Matrigel in mTeSR1, were used to form EBs in alpha-MEM (Life Technologies) containing 2 mM l-glutamine and 20% FBS. Medium was exchanged by sedimenting the EBs after 2 days and after 4 days the EBs were plated in 20% FBS/ alpha-MEM on tissue culture plasticware which had been coated overnight at 37 °C with 0.1% gelatin in dH_2_O. Culture medium was exchanged every 2-3 days.

### Karyotype and genotyping analysis

Performed by Cell Line Genetics, Madison, Wisconsin, USA.

### Nucleic acid extractions

Genomic DNA was extracted using the Wizard Genomic DNA Purification Kit (Promega) according to the manufacturer’s instructions.

Total RNA was extracted using a modification of the basic Trizol (Life Technologies) protocol. Briefly, 1 ml of Trizol was added to the sedimented colonies or EBs and triturated to dissociate the cells. At this point the lysates were stored at −80 °C until all samples for that cell line were collected. Upon thaw, lysates were incubated at room temperature for 10 min, mixed with 200 μl chloroform and centrifuged in a Phase-Lock Gel (Heavy) Eppendorf tube (Qiagen). RNA was precipitated from the aqueous phase by the addition of 250 μl of isopropanol and 250 μl of a high salt buffer (0.8 M sodium citrate and 1.2 M NaCl) followed by centrifugation. The RNA pellet was washed twice with 75% ethanol, dried and resuspended in nuclease-free water. RNA was DNase treated for 20 min and the DNAse removed using Ambion’s DNA-Free kit. Concentration was determined using a NanoDrop ND-1000 UV-VIS Spectrophotometer.

### PCR analysis of genomic DNA

PCR amplification of the viral sequences (WPRE) and GAPDH housekeeping gene was performed essentially according to the Millipore StemCCA instructions using AmpliTaq DNA Polymerase and GeneAmp dNTPs (Life Technologies). Custom primers were obtained from Invitrogen and 200 ng of genomic DNA was amplified using a GeneAmp 9600 according to the parameters: initial denaturation at 95 °C for 2 min, 30 cycles of 95 °C for 30 s, 65 °C for 45 s, 72 °C for 45 s followed by a 10 min extension at 72 °C. Products were visualized on a 2% agarose gel by standard gel electrophoresis.

### Gene expression microarray and statistical analysis of data

Global gene expression microarray was performed and analyzed using Agilent software, reagents and human One Color Gene Expression Oligo arrays according to the manufacturer’s instructions. The statistical programming language R (http://cran.r-project.org/) was used. Raw expression measurements for all gene probes for all samples were log (base = 2) transformed then quantile normalized. Quality of data was assured via sample-level inspection by Tukey box plot, covariance-based PCA scatter plot and correlation-based Heat Map. Raw expression measurements for samples deemed outliers were discarded and quantile normalization repeated. Gene probes not having at least one expression measurement greater than system noise post normalization were deemed “noise-biased” and discarded. System noise was defined as the lowest observed expression value at which the LOWESS (locally weighted scatterplot smoothing) fit of the data (CV ~ mean) for each class of samples (i.e., “hESC_undiff”, “hESC EB_ecto”, “hESC EB_mesend”, “hiPSC_undiff”, “hiPSC EB_ecto”, “hiPSC EB_mesend”) grossly deviates from linearity. For gene probes not discarded, expression measurements were floored to equal system noise if less than system noise then subject to ANOVA (analysis of variance) testing under BH (Benjamini and Hochberg) FDR (false discovery rate) MCC (multiple comparison correction) condition. Gene probes found to have a corrected P-value < 0.05 were deemed “potentially informative” and subject to the Tukey HSD (honestly significant difference) post-hoc test. Gene probes having a post-hoc P-value < 0.05 and a difference of class means ≥ 1.50 were deemed to have expression “significantly different” between the two classes. Annotation of these gene probes was accomplished using IPA (Ingenuity, Inc.).

### Methylation microarray and data analysis

Bisulfite conversion and array hybridization for methylation analysis was performed by MD Anderson Cancer Center, TX. Available average beta values were downloaded from NCBI GEO (http://www.ncbi.nlm.nih.gov/geo/query/acc. cgi?acc=GSE34869) and compared at the sample-level using Pearson correlation.

All microarray and methylation array data may be accessed through the NCBI GEO public database (Superseries number GSE51748).

## Results

### Derivation and characterization of H1-derived NPCs

**Figure 1:**
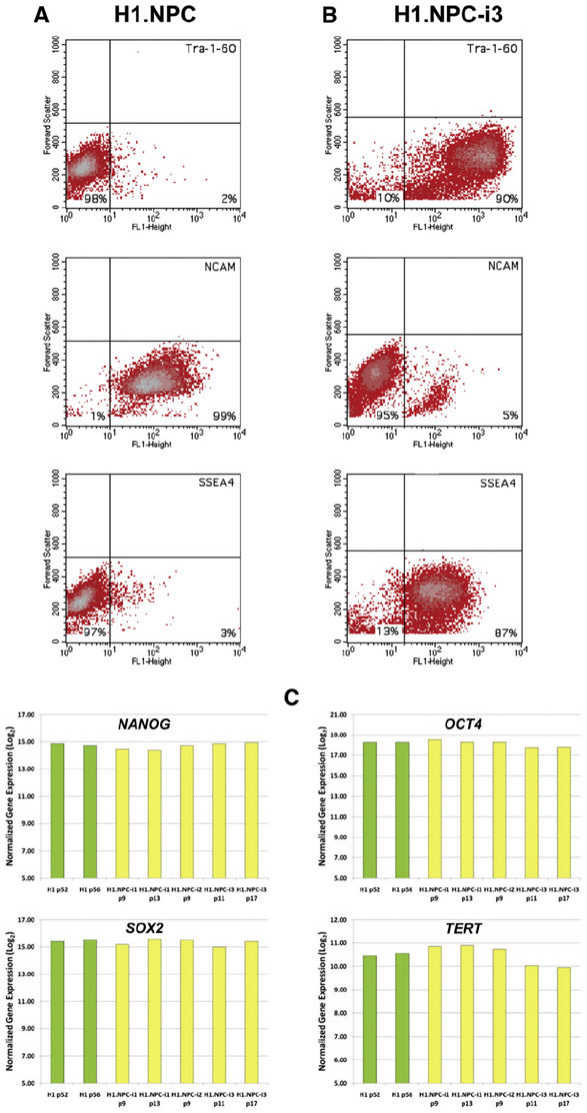
Characterization of the intermediate neural precursor (NPC) population and iPSCs. A and B) FACS analysis, represented by density plots of fluorescence intensity vs. forward scatter; A) Analysis of the intermediate neural precursor line, H1.NPC, shows the cells to be negative for the pluripotent stem cell markers, SSEA4 and Tra-1-60, and positive for the neural marker, polysialic neural cell adhesion molecule (NCAM). B) Analysis of the induced pluripotent stem cell line, H1.NPC-i3, shows the cells to be positive for SSEA4 and Tra-1-60 and negative for NCAM. C) Gene expression microarray analysis of NANOG, OCT4, SOX2 and TERT in H1 cells and derivatives showing similar levels of expression (log_2_ transformed quantile normalized) in both hESCs and hiPSCs. Embryonic stem cell samples are shown in green and iPSCs in yellow.

A population of expandable neuronal precursor cells (NPCs) were derived from the human embryonic stem cell line, H1 (WA01) as previously described (Kozhich et al., 2012). This cell line, designated H1.NPC, was negative for the hESC markers, SSEA4 and Tra-1-60 and positive for the neuronal marker, NCAM (Fig. 1A) by FACS analysis. Immunostaining showed the cells to be positive for nestin and TuJ1 indicating a mixed population of neural stem cells and neurons (Supplementary Fig. 1). At p2, H1.NPCs were subjected to negative magnetic sorting using Tra-1-60 antibody to remove any remaining pluripotent cells.

### Reprogramming-iPSC characterization

**Figure 2:**
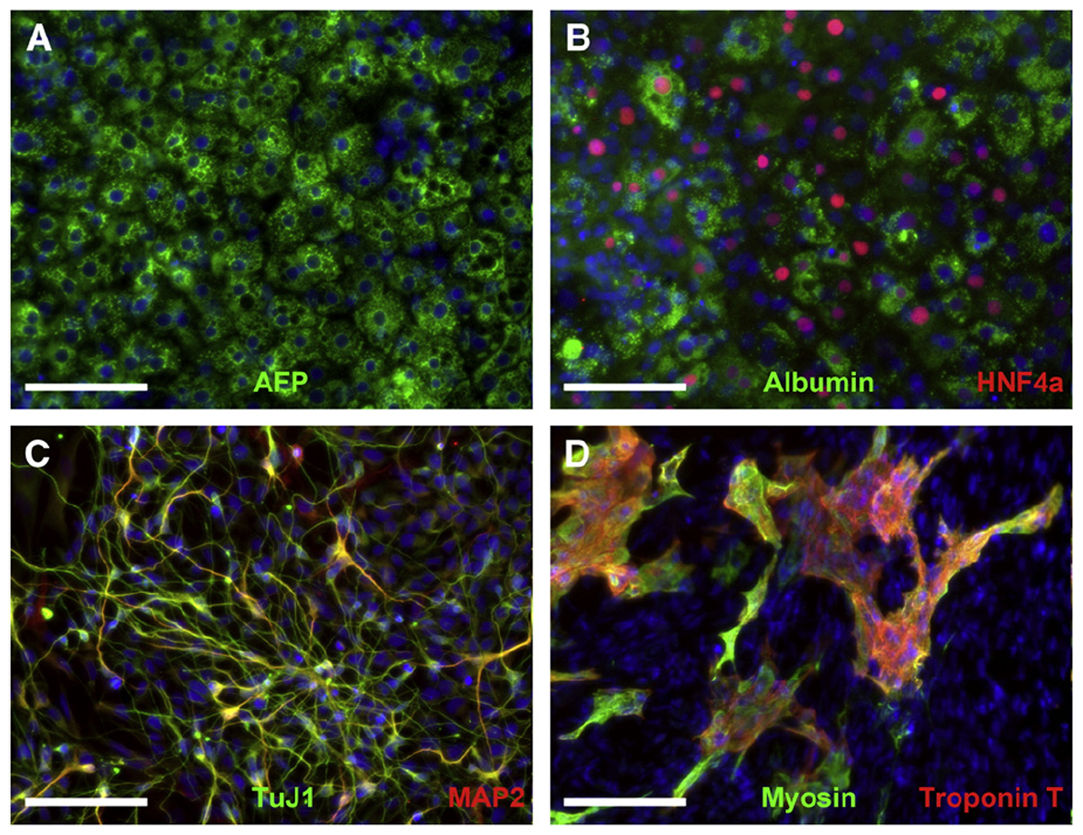
In vitro pluripotency assays for iPSC line, H1.NPC-i3. A & B) Endoderm differentiation – A) Directed differentiation generates cells positive for the hepatocyte markers, albumin (green) and HNF4α (red) as well as for alpha-fetoprotein (AFP) in B). C) Ectoderm differentiation – Neurons derived from H1.NPC-i3 are immunopositive for TuJ1 (green) with many MAP-2 positive cells (red). D) Mesoderm differentiation-cardiomyocytes are immunopositive for myosin heavy chain (green) and Troponin T (red). Note: Beating cardiomyocytes, indicative of mesoderm differentiation can also be viewed in Supplementary Video 1. Scale bar = 100 μm.

At p5, H1.NPCs were reprogrammed using the Millipore StemCCA lentiviral kit and plated out on MEF feeders in hESC medium. A total of 24 colonies were individually isolated directly from the feeder well and expanded on feeders. Remaining colonies were collected by collagenase digestion and replated on Matrigel. From this plate a further 12 colonies were individually isolated and expanded on Matrigel. Of these isolated colonies, 3 from each culture condition were analyzed by FACS for SSEA4, Tra-1-60 and NCAM. All cell lines were found to express SSEA4 and Tra-1-60, were immunonegative for SSEA1 and NCAM, as determined by FACS analysis, and were immunopositive for Oct4 (Fig. 1B and Supplementary Fig. 2). The three feeder-based iPSC lines, designated H1.NPC-i1, -i2 and -i3, were confirmed by STR analysis to have an identical genetic profile to the parent H1 line and had a normal karyotype at p14, p16 and p18 respectively (Supplementary Fig. 3). Each of these lines also exhibited a PCR product indicative of the presence of reprogramming viral sequences not present in the parent lines or neural precursor intermediate (Supplemental Fig. 4). By gene expression microarray analysis, the iPSC lines were shown to express the pluripotency genes, *NANOG* and *OCT4,* as well as telomere reverse transcriptase (*TERT*), at similar levels to hESC (Fig. 1C). These levels were reduced in the NPC population. *SOX2,* which is also present in neural precursors, is highly expressed in all cells (Fig. 1C). Two lines, H1.NPC-i1 and H1.NPC-i3, were tested for in vitro differentiation potential and were successfully directed to differentiate into cells representative of the 3 germ lineages. This is represented by Fig. 2 showing data for H1.NPC-i3 differentiation. Endoderm differentiation is demonstrated by immunoreactivity to AFP (Fig. 2A) as well as to HNF4α and albumin (Fig. 2B; hepatocytes), ectoderm differentiation by nestin and TuJ1 immunoreactivity (Fig. 2C; neurons) and mesoderm differentiation by myosin heavy chain β and troponin T immunoreactivity (Fig. 2D; cardiomyocytes) as well as by patches of spontaneously beating cells (Supplementary Video 1).

### Gene expression and methylation profiles of isogenic samples

**Figure 3:**
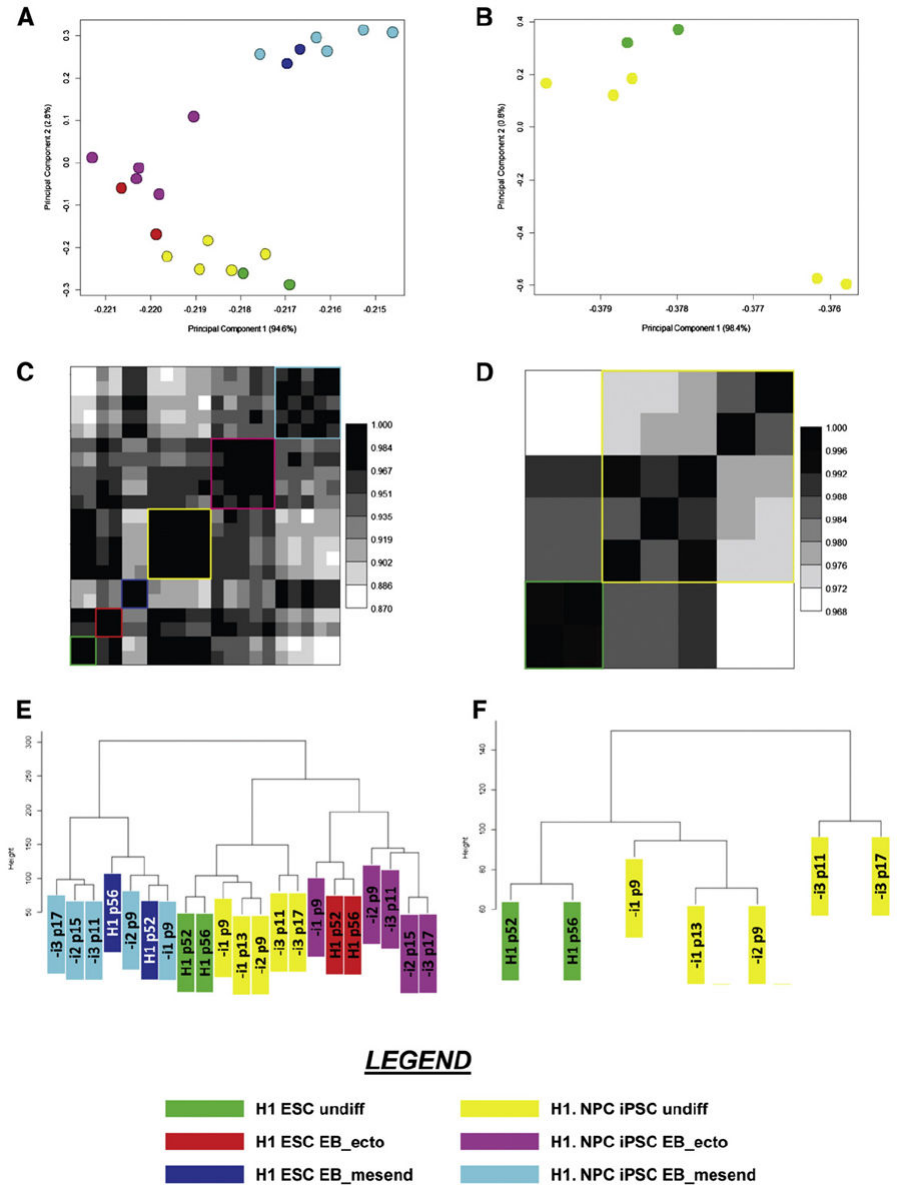
Gene expression array analysis. For all panels, log_2_ transformed quantile normalized Agilent expression values for a set of 38,449 gene probes, representing all probes expressed above noise, were used. Color-coding is uniform across all panels and is explained in the legend. H1 ESC undiff – green; H1.NPC iPSC undiff – yellow; H1 ESC EB_ecto – red; H1.NPC iPSC EB_ecto – magenta; H1 ESC EB_mesend – blue; H1.NPC iPSC EB_mesend – cyan. (A & B) Covariance PCA Scatterplot. A) 21 undifferentiated and differentiated samples. The x-axis describes the percent total variance explained by the first principal component (94.6%). The y-axis describes the percent total variance explained by the second principal component (2.8%). B) 7 undifferentiated samples. The x-axis describes the percent total variance explained by the first principal component (98.4%). The y-axis describes the percent total variance explained by the second principal component (0.8%). (C & D) Pearson Correlation Heat Map. C) Map depicts 21 undifferentiated and differentiated samples. D) Map depicts 7 undifferentiated samples. (E & F) Hierarchical Cluster Dendrogram. E) Dendrogram depicts 21 undifferentiated and differentiated samples. F) Dendrogram depicts 7 undifferentiated samples. For all images, the statistical programming language R was used (www.http://www.r-project.org/).

Since our original gene expression database, StemCellDB, was established using MEF-based cultures (Mallon et al., 2013) we chose the 3 iPSC lines derived on MEFs for further analysis. Samples were taken from each line for RNA extraction, gDNA extraction, FACS analysis and embryoid body formation in 2 media that had been shown to direct cells toward a mesendodermal lineage (EB_mesend) or an ectodermal lineage (EB_ecto). After 8 days in culture total RNA was extracted from the embryoid bodies. RNA from all undifferentiated and differentiated cells, including H1 hESCs, were analyzed by Agilent One-color microarray. Bioinformatics analysis revealed no major difference in global gene expression pattern between the hESC and hiPSC populations under any culture condition (Figs. 3A, C and E). Notably, analysis of the undifferentiated samples indicates that there appears to be more variability between individual samples than between the two populations (Figs. 3B, D and F). However, using a 1.5-fold threshold, 625 gene probes were found to be statistically differentially expressed between the undifferentiated populations (Supplementary Table 1). This was the lowest number of differentially expressed probes found between any pairwise comparison and most appear to be minor variations, reflecting the high overall similarity of the populations. The number of called probes drops to 260 when a 2-fold threshold is used. In addition, testing of the undifferentiated sample classes directly using the Welch-modified *t*-test under corrected conditions identifies no gene as being differentially expressed between hESC and iPSC when a fold-difference magnitude of 2 is used. Upon examination of many of the genes described in the previous study as being differentially expressed between isogenic hESCs and hiPSCs (Teichroeb et al., 2011), we found no significant difference in expression in our isogenic samples (Supplementary Fig. 5). However, many of these genes were found to be variably expressed in the undifferentiated state or upon differentiation, which may contribute to the observation of differences in the nominally undifferentiated state depending on the quality of the culture.

The methylation profiles of undifferentiated H1 ESC and iPSC lines as well as H1.NPC were analyzed by bisulfate conversion and array hybridization. We found that the pluripotent cell populations exhibited 98–99% accordance with each other but only 92–93% accordance with the NPC population (Table 1).

All microarray and methylation array data may be accessed through the NCBI GEO public database (Superseries number GSE51748).

### Regulation of NNAT by methylation

**Figure 4:**
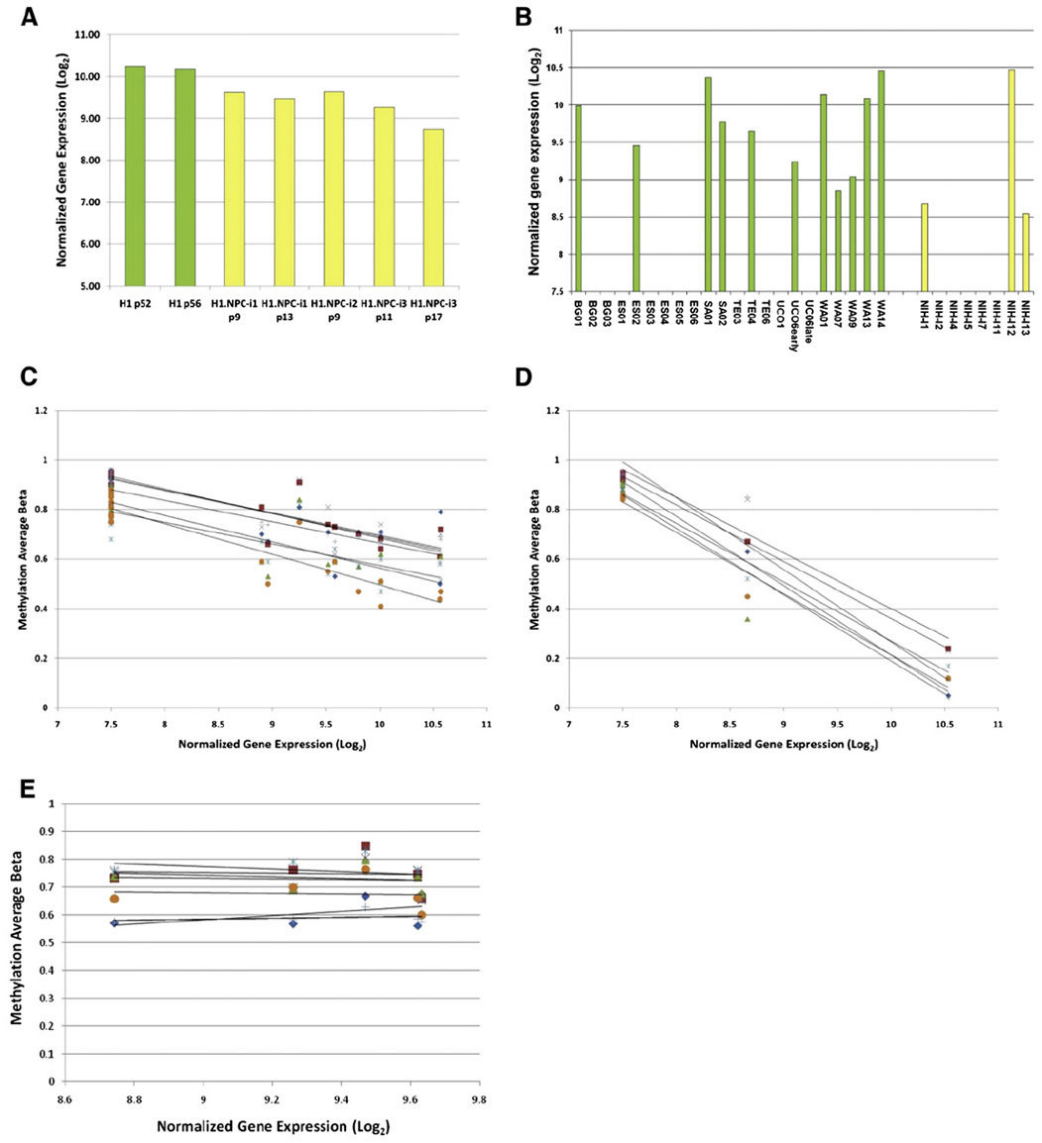
Regulation of *NNAT* gene expression. A) *NNAT* gene expression in H1 and derivatives shows little variation in isogenic pluripotent stem cell populations. B) *NNAT* gene expression in 21 genetically unique hESC and 8 hiPSC lines (StemCellDB) showing variability in expression levels. For both A) and B), undifferentiated hESCs are shown in green and hiPSCs in yellow. (C and D) Gene expression of *NNAT* in StemCellDB samples is reduced with increasing methylation but the decrease in expression is more profound in hiPSCs (C) than in hESCs (D). E) Isogenic H1 hiPSCs show little effect of methylation.

In the previous isogenic H9 hESC vs. hiPSC study, the only gene that showed significant variation was neonatin (Teichroeb et al., 2011). In contrast, *NNAT* does not meet the criteria for significantly different gene expression levels between the isogenic H1 hESCs and their hiPSC derivatives (Fig. 4A). However, upon examination of StemCellDB it is clear that *NNAT* expression varies considerably within both hESC and hiPSC populations (Fig. 4B). We correlated methylation to gene expression levels in the StemCellDB samples and confirm that gene expression is reduced with increasing methylation as previously described. Interestingly, in StemCellDB, it appears that the effect of methylation on gene expression levels is much more profound in hiPSCs than in the hESCs where the average r^2^ values for all methylation probes are 0.961 and 0.766 respectively (Figs. 4C and D). However, although there is some variability in gene expression and methylation levels in the current isogenic H1 hiPSC samples, the correlation is not striking. In addition, with only 2 samples, it is not statistically relevant to obtain an r^2^ value for the H1 hESCs.

## Discussion

Although the molecular profiles of many isogenic cells have been compared, these studies mostly refer to differentiated cells, such as blood cells, used to derive iPSCs, which have been subsequently differentiated back into blood (Kim et al., 2010). The first comparison of isogenic pluripotent cells was conducted with the female H9 hESC line and indicated that there was significant variation between hESCs and hiPSCs in the expression of neonatin–*NNAT* (Teichroeb et al., 2011). This group also found much clonal variation in the expression of several imprinted genes and concluded that reprogramming can lead to dysregulation of genomic imprinting. In contrast, we find no significant difference in the expression of *NNAT* in the current samples derived from the H1 hESC line. However, examination of our publicly available database, StemCellDB (Mallon et al., 2013), shows that *NNAT* is variably expressed in 21 genetically unique hESCs and 8 hiPSCs. As demonstrated by Teichroeb et al, gene expression of *NNAT* is modulated by methylation in these samples but, interestingly, it may be that hiPSCs are more sensitive to this regulation. In many cases, gene expression varies as a function of differentiation and so variable expression of genes described by Teichroeb et al. (2011) and others may well be due to alteration of culture conditions during reprogramming and in routine maintenance of the cells (Newman and Cooper, 2010; Wutz, 2012).

In this study, we find few gene expression differences in any undifferentiated or differentiated condition in accordance with our previous findings (Mallon et al., 2013). We also find no global differences in the methylation profile of the pluripotent populations. Although our findings on isogenic cells varied from that of Teichroeb et al., it could be that XCI effects are responsible for the variations observed in the female, H9, derivatives which are not apparent in the male, H1, derivatives. Interestingly, although we did not find variation in *NNAT* expression in our isogenic lines, we did find variability in a larger population of cells which was inversely correlated with methylation. This correlation was more profound in hiPSCs than in hESCs perhaps suggesting that hiPSCs have a more open genomic conformation or that *NNAT* is exclusively regulated by methylation in the iPSCs whereas other elements can influence its expression in the hESCs. However, this profound correlation was not observed in the current hiPSC samples suggesting that this regulation is either genome-specific or is possibly related to the method of reprogramming. The original samples were reprogrammed with individual retroviruses whereas the current samples were reprogrammed using a polycistronic lentivirus. Further studies should be able to clarify this point.

Both gene expression and methylation analyses revealed differences between the pluripotent cells and the intermediate NPCs. However, it would appear that epigenetic memory is not grossly apparent in the hiPSC lines at the methylation level at the passages tested. Since some of these passages are relatively early (p9) this may contrast with previous studies (Kim et al., 2010) but it should be emphasized that the differentiated cell population used in this study was derived from pluripotent cells and not tissue. Therefore, it is possible that not all epigenetic marks of the pluripotent state had been erased in the intermediate population. There appears to be mounting evidence, however, that epigenetic changes observed between hESCs and hiPSCs may be due in large part to culture conditions (Newman and Cooper, 2010; Wutz, 2012). This could include not only the local culture maintenance protocols, but the vector(s) used as well as the manner of reprogramming itself. Further studies of this type should help to elucidate and understand the mechanism of such epigenetic drift. In conclusion, our findings show that reprogramming itself does not inherently affect the genetic or epigenetic profile of the cells which is important as we move iPSCs toward drug development or therapeutic application.

## Supplementary Material

Supplementary Figures 1-5

Supplementary Table 1

Supplementary Video 1

## Figures and Tables

**Table 1:** Cross-sample Comparison of Methylation Patterns. The pair-wise comparison of 27,578 average beta values using Pearson correlation is depicted. Values represented in the table are the resulting correlation coefficients (Pearson’s R) and have been shaded to reflect pair-wise relationships with perfect positive relationships between samples (Pearson R = 1.00) color-coded dark orange. R values less than perfect are colored lighter orange with lowest correlations not colored.

Methylation	H1 p52	H1 p56	H1.NPC p13	H1.NPC-i1 p9	H1.NPC-i1 p13	H1.NPC-i2p9	H1.NPC-i2p15	H1.NPC-i3 p11	H1.NPC-i3 p17
H1 p52	1.00								
H1 p56	1.00	1.00							
H1.NPC p13	0.93	0.92	1.00						
H1.NPC-i1 p9	0.99	0.99	0.93	1.00					
H1.NPC-i1 p13	0.99	0.99	0.93	0.99	1.00				
H1.NPC-i2 p9	0.99	0.99	0.93	0.99	0.99	1.00			
H1.NPC-i2 p15	0.98	0.99	0.92	0.98	0.99	0.99	1.00		
H1.NPC-i3 p11	0.98	0.98	0.93	0.99	0.99	0.99	0.98	1.00	
H1.NPC-i3 p17	0.99	0.99	0.93	0.99	0.99	0.99	0.98	1.00	1.00

## References

[R1] Bock C, Kiskinis E, Verstappen G, Gu H, Boulting G, Smith ZD, Ziller M, Croft GF, Amoroso MW, Oakley DH (2011). Reference maps of human ES and iPS cell variation enable high-throughput characterization of pluripotent cell lines. Cell.

[R2] Chin MH, Mason MJ, Xie W, Volinia S, Singer M, Peterson C, Ambartsumyan G, Aimiuwu O, Richter L, Zhang J (2009). Induced pluripotent stem cells and embryonic stem cells are distinguished by gene expression signatures. Cell Stem Cell.

[R3] Deng J, Shoemaker R, Xie B, Gore A, LeProust EM, Antosiewicz-Bourget J, Egli D, Maherali N, Park IH, Yu J (2009). Targeted bisulfite sequencing reveals changes in DNA methylation associated with nuclear reprogramming. Nat Biotechnol.

[R4] Doi A, Park IH, Wen B, Murakami P, Aryee MJ, Irizarry R, Herb B, Ladd-Acosta C, Rho J, Loewer S (2009). Differential methylation of tissue- and cancer-specific CpG island shores distinguishes human induced pluripotent stem cells, embryonic stem cells and fibroblasts. Nat Genet.

[R5] Ebert AD, Yu J, Rose FF, Mattis VB, Lorson CL, Thomson JA, Svendsen CN (2009). Induced pluripotent stem cells from a spinal muscular atrophy patient. Nature.

[R6] Feng Q, Lu SJ, Klimanskaya I, Gomes I, Kim D, Chung Y, Honig GR, Kim KS, Lanza R (2010). Hemangioblastic derivatives from human induced pluripotent stem cells exhibit limited expansion and early senescence. Stem Cells.

[R7] Gore A, Li Z, Fung HL, Young JE, Agarwal S, Antosiewicz-Bourget J, Canto I, Giorgetti A, Israel MA, Kiskinis E (2011). Somatic coding mutations in human induced pluripotent stem cells. Nature.

[R8] Guenther MG, Frampton GM, Soldner F, Hockemeyer D, Mitalipova M, Jaenisch R, Young RA (2010). Chromatin structure and gene expression programs of human embryonic and induced pluripotent stem cells. Cell Stem Cell.

[R9] He JQ, Ma Y, Lee Y, Thomson JA, Kamp TJ (2003). Human embryonic stem cells develop into multiple types of cardiac myocytes: action potential characterization. Circ Res.

[R10] Hu BY, Weick JP, Yu J, Ma LX, Zhang XQ, Thomson JA, Zhang SC (2010). Neural differentiation of human induced pluripotent stem cells follows developmental principles but with variable potency. PNAS USA.

[R11] Hussein SM, Batada NN, Vuoristo S, Ching RW, Autio R, Narva E, Ng S, Sourour M, Hamalainen R, Olsson C (2011). Copy number variation and selection during reprogramming to pluripotency. Nature.

[R12] Kim K, Doi A, Wen B, Ng K, Zhao R, Cahan P, Kim J, Aryee MJ, Ji H, Ehrlich LI (2010). Epigenetic memory in induced pluripotent stem cells. Nature.

[R13] Kozhich OA, Hamilton RS, Mallon BS (2012). Standardized generation and differentiation of neural precursor cells from human pluripotent stem cells. Stem Cell Rev Rep.

[R14] Laurent LC, Ulitsky I, Slavin I, Tran H, Schork A, Morey R, Lynch C, Harness JV, Lee S, Barrero MJ (2011). Dynamic changes in the copy number of pluripotency and cell proliferation genes in human ESCs and iPSCs during reprogramming and time in culture. Cell Stem Cell.

[R15] Lister R, Pelizzola M, Dowen RH, Hawkins RD, Hon G, Tonti-Filippini J, Nery JR, Lee L, Ye Z, Ngo QM (2009). Human DNA methylomes at base resolution show widespread epigenomic differences. Nature.

[R16] Lister R, Pelizzola M, Kida YS, Hawkins RD, Nery JR, Hon G, Antosiewicz-Bourget J, O’Malley R, Castanon R, Klugman S (2011). Hotspots of aberrant epigenomic reprogramming in human induced pluripotent stem cells. Nature.

[R17] Lo HS, Wang Z, Hu Y, Yang HH, Gere S, Buetow KH, Lee MP (2003). Allelic variation in gene expression is common in the human genome. Genome Res.

[R18] Mallon BS, Chenoweth JG, Johnson KR, Hamilton RS, Tesar PJ, Yavatkar AS, Tyson LJ, Park K, Chen KG, Fann YC (2013). StemCellDB: the human pluripotent stem cell database at the National Institutes of Health. Stem Cell Res.

[R19] Marchetto MC, Yeo GW, Kainohana O, Marsala M, Gage FH, Muotri AR (2009). Transcriptional signature and memory retention of human-induced pluripotent stem cells. PLoS One.

[R20] Mayshar Y, Ben-David U, Lavon N, Biancotti JC, Yakir B, Clark AT, Plath K, Lowry WE, Benvenisty N (2010). Identification and classification of chromosomal aberrations in human induced pluripotent stem cells. Cell Stem Cell.

[R21] Newman AM, Cooper JB (2010). Lab-specific gene expression signatures in pluripotent stem cells. Cell Stem Cell.

[R22] Si-Tayeb K, Noto FK, Nagaoka M, Li J, Battle MA, Duris C, North PE, Dalton S, Duncan SA (2010). Highly efficient generation of human hepatocyte-like cells from induced pluripotent stem cells. Hepatology.

[R23] Smith KP, Luong MX, Stein GS (2009). Pluripotency: toward a gold standard for human ES and iPS cells. J Cell Physiol.

[R24] Soldner F, Hockemeyer D, Beard C, Gao Q, Bell GW, Cook EG, Hargus G, Blak A, Cooper O, Mitalipova M (2009). Parkinson’s disease patient-derived induced pluripotent stem cells free of viral reprogramming factors. Cell.

[R25] Teichroeb JH, Betts DH, Vaziri H (2011). Suppression of the imprinted gene NNAT and X-chromosome gene activation in isogenic human iPS cells. PLoS One.

[R26] Wutz A (2012). Epigenetic alterations in human pluripotent stem cells: a tale of two cultures. Cell Stem Cell.

[R27] Yan H, Yuan W, Velculescu VE, Vogelstein B, Kinzler KW (2002). Allelic variation in human gene expression. Science.

[R28] Zhu S, Li W, Zhou H, Wei W, Ambasudhan R, Lin T, Kim J, Zhang K, Ding S (2010). Reprogramming of human primary somatic cells by OCT4 and chemical compounds. Cell Stem Cell.

