# Supplementary material for: Comparison of the molecular profiles of human embryonic and induced pluripotent stem cells of isogenic origin
Source: Stem Cell Res. Author manuscript; Available in PMC 2015 Mar 1. (PMC4157340; doi:10.1016/j.scr.2013.11.010)
Supplement: Supplementary Figures 1-5 [file NIHMS620860-supplement-Supplementary_Figures_1-5.ppt]

## Slide 1
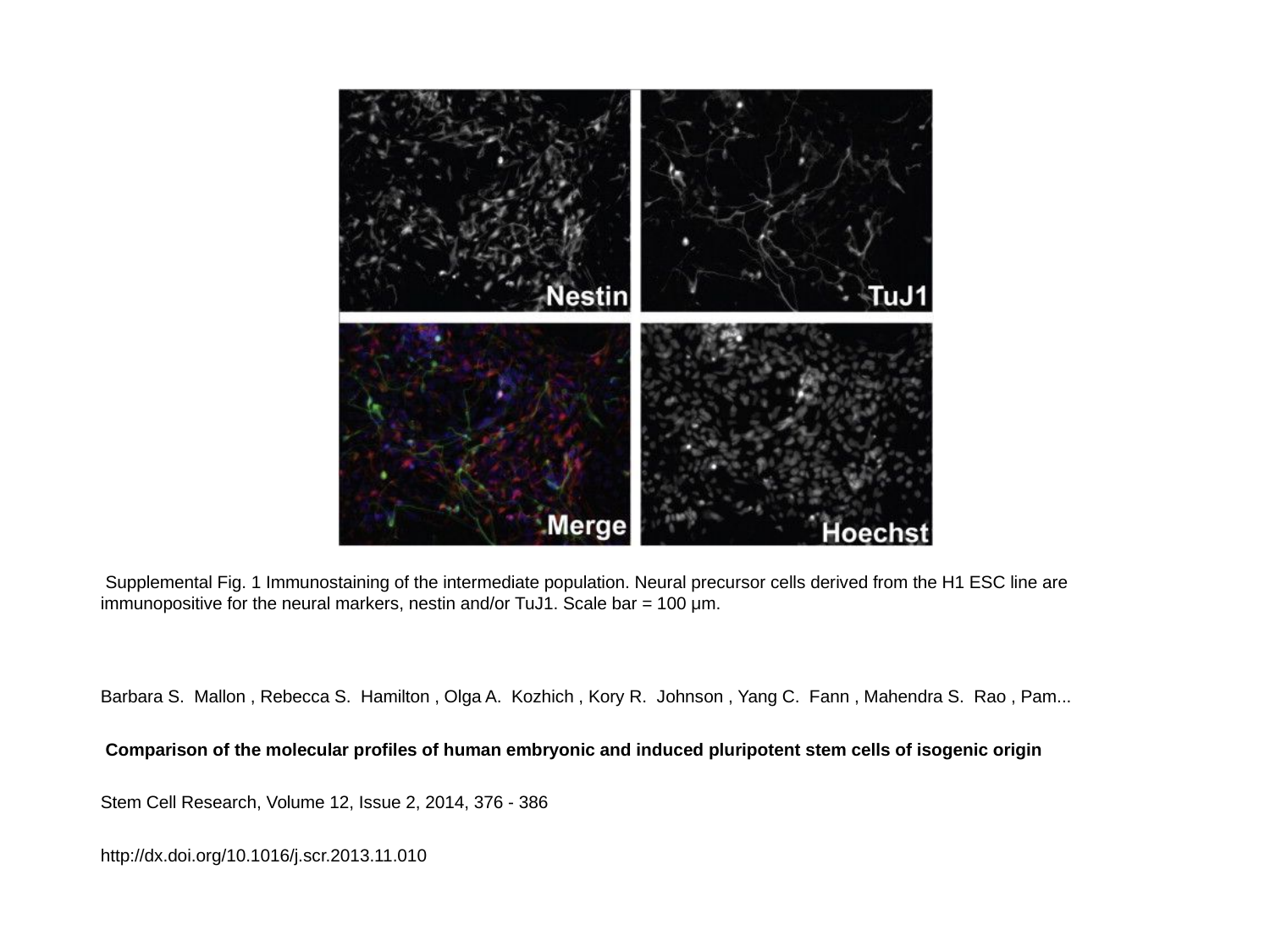

Supplemental Fig. 1 Immunostaining of the intermediate population. Neural precursor cells derived from the H1 ESC line are immunopositive for the neural markers, nestin and/or TuJ1. Scale bar = 100 μm.
Barbara S. Mallon , Rebecca S. Hamilton , Olga A. Kozhich , Kory R. Johnson , Yang C. Fann , Mahendra S. Rao , Pam...
 Comparison of the molecular profiles of human embryonic and induced pluripotent stem cells of isogenic origin
Stem Cell Research, Volume 12, Issue 2, 2014, 376 - 386
http://dx.doi.org/10.1016/j.scr.2013.11.010

## Slide 2
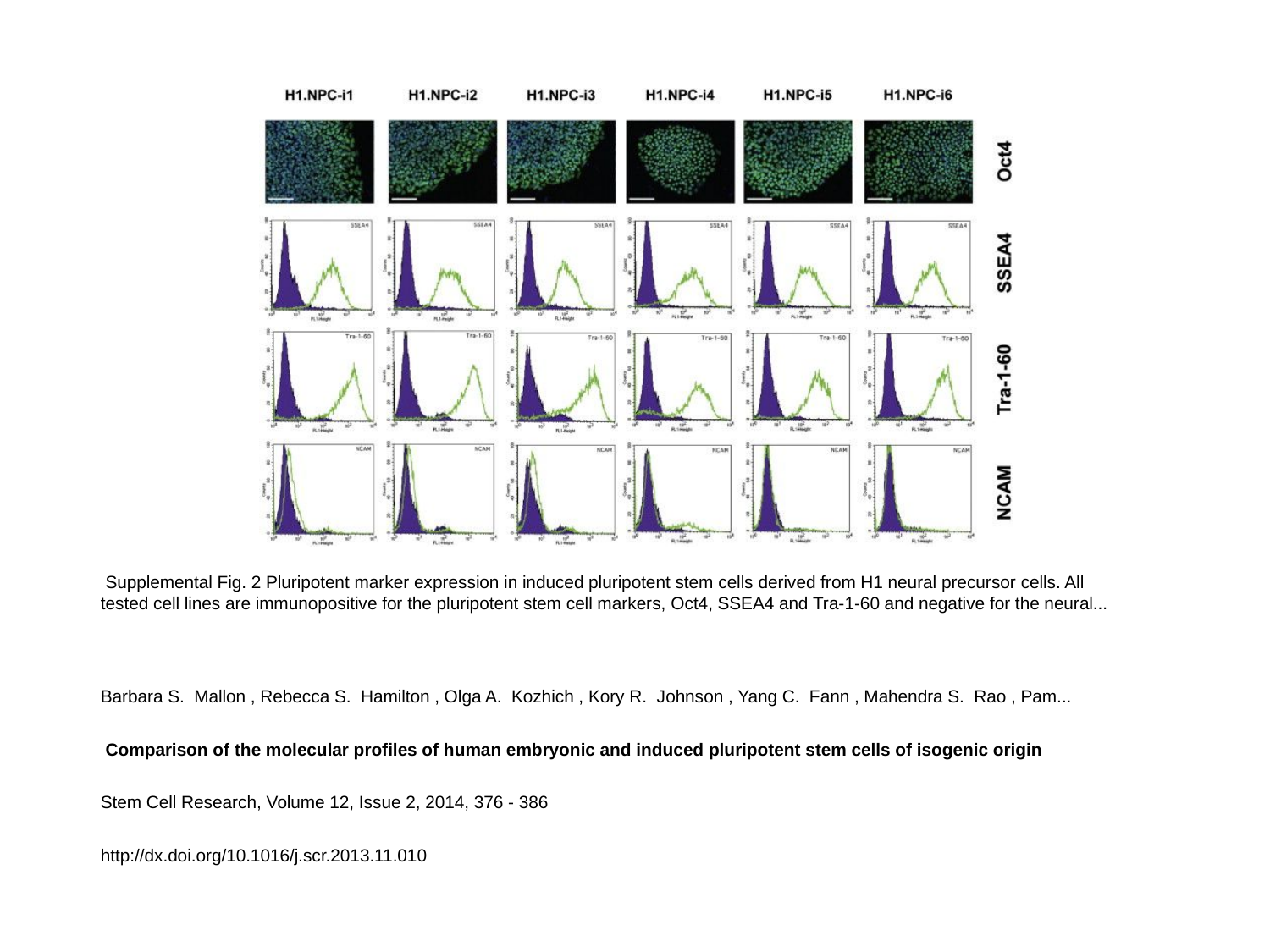

Supplemental Fig. 2 Pluripotent marker expression in induced pluripotent stem cells derived from H1 neural precursor cells. All tested cell lines are immunopositive for the pluripotent stem cell markers, Oct4, SSEA4 and Tra-1-60 and negative for the neural...
Barbara S. Mallon , Rebecca S. Hamilton , Olga A. Kozhich , Kory R. Johnson , Yang C. Fann , Mahendra S. Rao , Pam...
 Comparison of the molecular profiles of human embryonic and induced pluripotent stem cells of isogenic origin
Stem Cell Research, Volume 12, Issue 2, 2014, 376 - 386
http://dx.doi.org/10.1016/j.scr.2013.11.010

## Slide 3
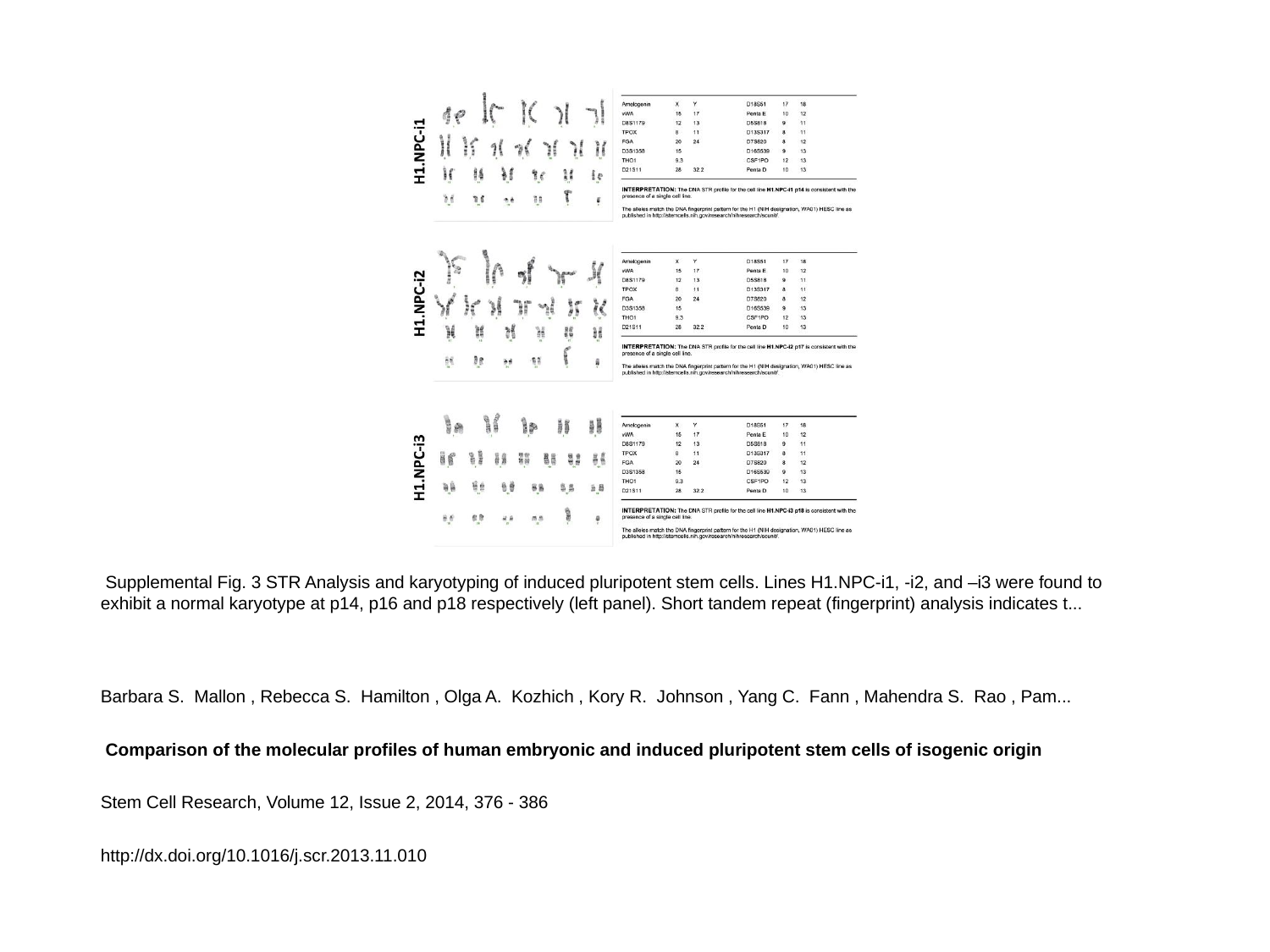

Supplemental Fig. 3 STR Analysis and karyotyping of induced pluripotent stem cells. Lines H1.NPC-i1, -i2, and –i3 were found to exhibit a normal karyotype at p14, p16 and p18 respectively (left panel). Short tandem repeat (fingerprint) analysis indicates t...
Barbara S. Mallon , Rebecca S. Hamilton , Olga A. Kozhich , Kory R. Johnson , Yang C. Fann , Mahendra S. Rao , Pam...
 Comparison of the molecular profiles of human embryonic and induced pluripotent stem cells of isogenic origin
Stem Cell Research, Volume 12, Issue 2, 2014, 376 - 386
http://dx.doi.org/10.1016/j.scr.2013.11.010

## Slide 4
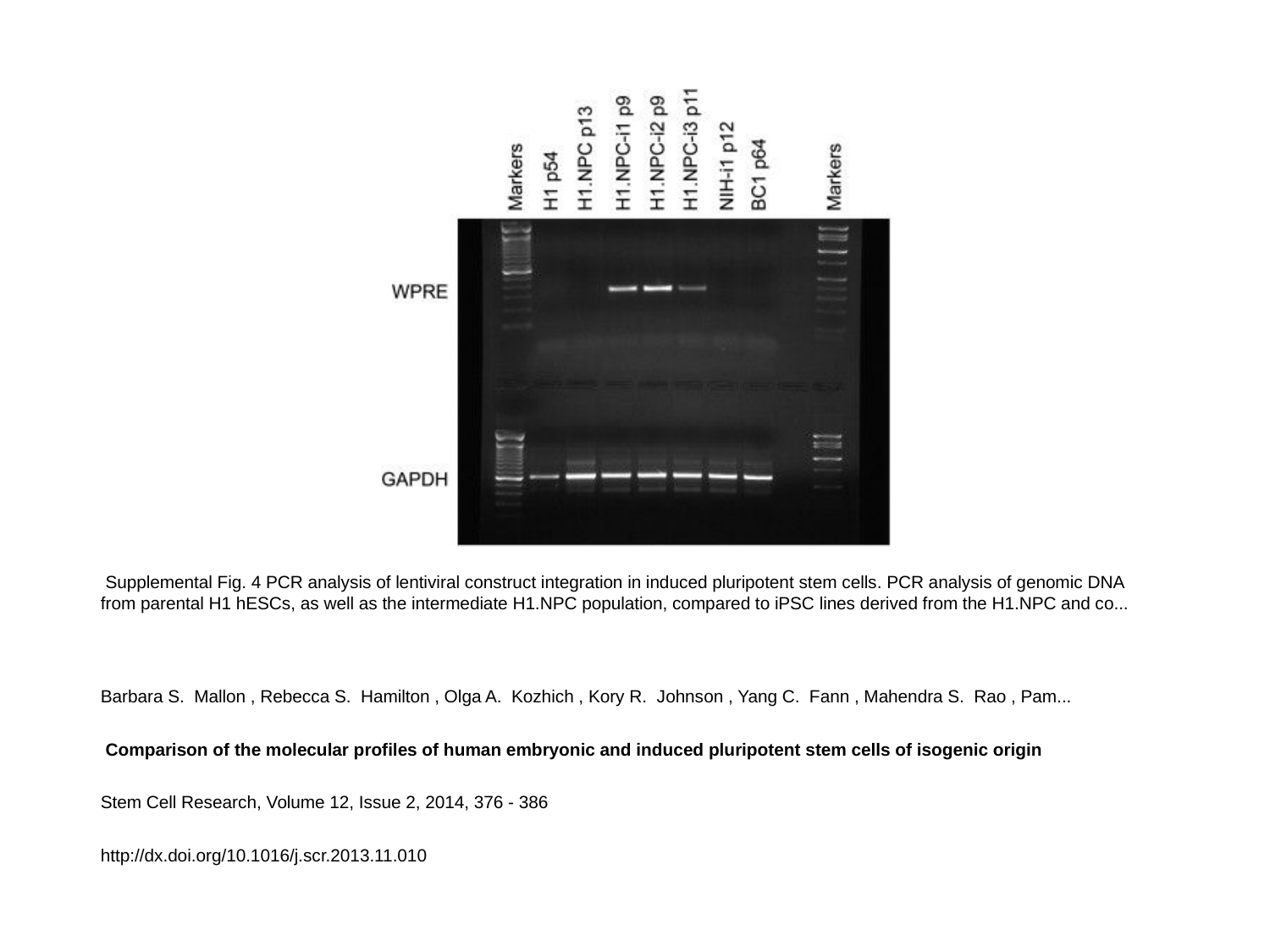

Supplemental Fig. 4 PCR analysis of lentiviral construct integration in induced pluripotent stem cells. PCR analysis of genomic DNA from parental H1 hESCs, as well as the intermediate H1.NPC population, compared to iPSC lines derived from the H1.NPC and co...
Barbara S. Mallon , Rebecca S. Hamilton , Olga A. Kozhich , Kory R. Johnson , Yang C. Fann , Mahendra S. Rao , Pam...
 Comparison of the molecular profiles of human embryonic and induced pluripotent stem cells of isogenic origin
Stem Cell Research, Volume 12, Issue 2, 2014, 376 - 386
http://dx.doi.org/10.1016/j.scr.2013.11.010

## Slide 5
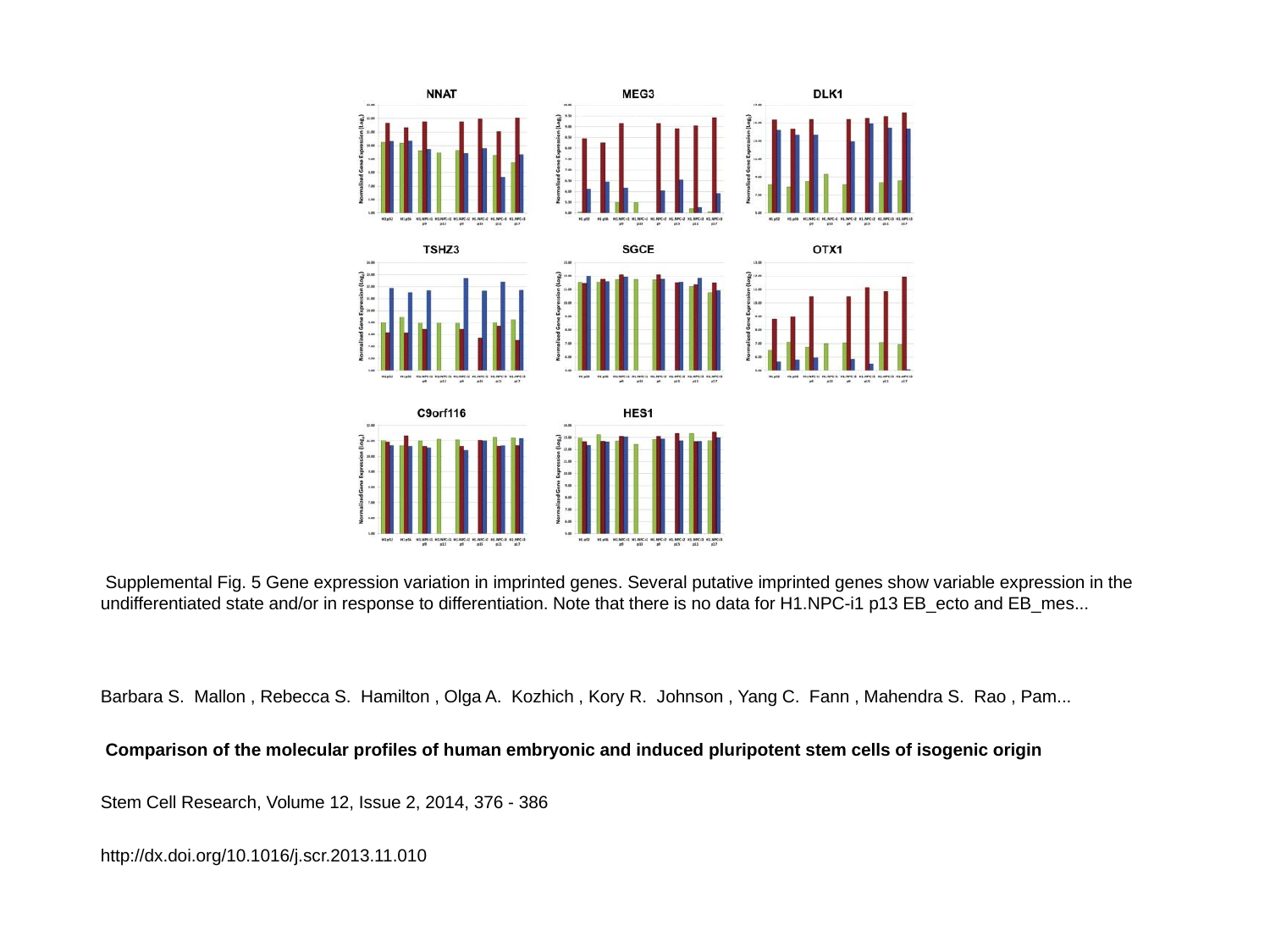

Supplemental Fig. 5 Gene expression variation in imprinted genes. Several putative imprinted genes show variable expression in the undifferentiated state and/or in response to differentiation. Note that there is no data for H1.NPC-i1 p13 EB_ecto and EB_mes...
Barbara S. Mallon , Rebecca S. Hamilton , Olga A. Kozhich , Kory R. Johnson , Yang C. Fann , Mahendra S. Rao , Pam...
 Comparison of the molecular profiles of human embryonic and induced pluripotent stem cells of isogenic origin
Stem Cell Research, Volume 12, Issue 2, 2014, 376 - 386
http://dx.doi.org/10.1016/j.scr.2013.11.010
